# Efficacy of Probiotic VITA-PB2 from Fermented Foods on Alcohol Consumption and Hangover Symptoms: A Randomized, Double-Blind, Placebo-Controlled Trial

**DOI:** 10.3390/nu17142276

**Published:** 2025-07-09

**Authors:** Chaodeng Mo, Johny Bajgai, Md. Habibur Rahman, Sofian Abdul-Nasir, Hui Ma, Thu Thao Pham, Haiyang Zhang, Buchan Cao, Seong Hoon Goh, Bomi Kim, Hongik Kim, Min Kyeong Seol, Young Geon Yu, Cheol-Su Kim, Kyu-Jae Lee, Seung-Taek Lim

**Affiliations:** 1 Department of Convergence Medicine, Wonju College of Medicine, Yonsei University, Wonju 26426, Republic of Korea; chaodengmo@gmail.com (C.M.); johnybajgai@gmail.com (J.B.); globaldreamer1990@gmail.com (M.H.R.); abdulnasirsofian62@gmail.com (S.A.-N.); mahui56@yonsei.ac.kr (H.M.); phamthuthaoytcc@gmail.com (T.T.P.); zhanghyzrc1017@gmail.com (H.Z.); caobuchan777@gmail.com (B.C.); forget419@yonsei.ac.kr (S.H.G.); kimbomi9090@gmail.com (B.K.); cs-kim@yonsei.ac.kr (C.-S.K.); 2 Department of Global Medical Science, Yonsei University Graduate School, Wonju College of Medicine, Wonju 26426, Republic of Korea; 3 Department of Laboratory Medicine, Wonju College of Medicine, Yonsei University, Wonju 26426, Republic of Korea; 4 Vitabio Inc., Sejong 30141, Republic of Korea; hikim@vitabio.net (H.K.); smk20@vitabio.net (M.K.S.); 5 Essobiome Inc., Sejong 30141, Republic of Korea; uygg2805@naver.com; 6 Department of Hematology-Oncology, Wonju College of Medicine, Yonsei University, Wonju 26426, Republic of Korea

**Keywords:** *Leuconostoc mesenteroides*, ethanol metabolism, acetaldehyde, aldehyde dehydrogenase, probiotics, oxidative stress, hangover

## Abstract

**Background**: Modulating ethanol metabolism and attenuating alcohol-induced oxidative stress are promising therapeutic strategies for reducing the severity of hangovers and alleviating their associated physiological burden. **Methods**: A randomized, double-blind, placebo-controlled, crossover study was conducted to evaluate the effects of the probiotic strain *Leuconostoc mesenteroides* VITA-PB2 on ethanol metabolism, oxidative stress, and hangover-related symptoms in 28 healthy adults. The participants consumed either VITA-PB2 or a placebo before standardized alcohol intake, with a 7-day washout period and subsequent crossover. Primary outcomes included blood ethanol, acetaldehyde levels, and aldehyde dehydrogenase (ALDH) activity. Secondary outcomes measured hangover severity assessed by the Acute Hangover Scale (AHS), liver enzymes including aspartate aminotransferase (AST), alanine aminotransferase (ALT), and gamma-glutamyl transferase (GGT), oxidative stress indicators reactive oxygen species (ROS) and nitric oxide (NO), and antioxidant responses measured by glutathione peroxidase (GPx), catalase, and 2,2-diphenyl-1-picrylhydrazyl (DPPH) scavenging capacity. **Results**: VITA-PB2 supplementation led to a sustained reduction in blood ethanol concentrations beginning at 0.5 h post-ingestion compared with the placebo group, indicating more efficient ethanol clearance. Additionally, VITA-PB2 significantly reduced acetaldehyde levels at 1 h post-ingestion (*p* < 0.05) and increased ALDH activity by 42.15% at 30 min (*p* < 0.05). It also markedly reduced ROS levels at 1 h (*p* < 0.05), enhanced glutathione peroxidase (GPx) activity at 2 h (*p* < 0.01), and significantly improved the subjective hangover symptoms, particularly thirst (*p* < 0.05). **Conclusions**: No adverse effects were reported during the trial, indicating that *Leuconostoc mesenteroides* VITA-PB2 is a safe probiotic. These findings suggest its efficacy in mitigating alcohol-induced oxidative stress and alleviating hangover-related symptoms.

## 1. Introduction

Alcohol consumption represents a critical global public health issue, exhibiting substantial variations in consumption patterns across diverse regions. Globally, the average annual consumption of pure ethanol is approximately 6.4 L [1]; however, consumption significantly exceeds this average in certain countries, surpassing 12 L per capita annually [2]. Such widespread alcohol intake imposes considerable economic burdens, primarily through increased healthcare expenditures, reduced productivity, and a higher incidence of alcohol-related accidents and chronic illnesses, including liver diseases, cardiovascular conditions, and neuropsychiatric disorders [3,4].

Approximately 90–98% of ethanol is absorbed in the gastrointestinal tract, predominantly through the stomach and small intestine [5]. Following absorption, ethanol is metabolized in the cytoplasm of hepatocytes by alcohol dehydrogenase (ADH) into acetaldehyde. Acetaldehyde is further converted into acetic acid by mitochondrial acetaldehyde dehydrogenase (ALDH), ultimately entering energy metabolism pathways [6]. Acetaldehyde, as the primary toxic intermediate metabolite, induces various adverse physiological effects, including headache, facial flushing, tachycardia, nausea, vomiting, and significant systemic toxicity [7,8].

Hangovers, characterized by a spectrum of physiological and psychological discomfort, commonly occur following excessive alcohol consumption and markedly impair cognitive performance and daily activities. Typical symptoms include headache, fatigue, myalgia, gastrointestinal disturbances, thirst, and psychological manifestations, such as anxiety and depression [9,10]. The pathophysiology of hangovers is multifaceted and involves cytotoxicity due to acetaldehyde accumulation, oxidative stress, systemic inflammation, and gut microbiota dysbiosis [11,12]. Notably, alterations in the composition of the gut microbiota have been increasingly recognized as crucial contributors to the severity of hangovers. Emerging evidence from randomized controlled trials suggests that probiotics and prebiotics effectively mitigate hangover symptoms by modulating the gut microbial balance [13]. *Leuconostoc* spp., which are widely recognized as probiotics, have gained attention for their ability to metabolize ethanol and detoxify acetaldehyde [14]. These probiotics also exhibit antioxidant and anti-inflammatory properties, enhance intestinal barrier function via exopolysaccharide production, and reduce endotoxin translocation [15]. Emerging evidence suggests their potential neuroprotective and anti-intracellular stress effects [16].

Among the lactobacilli, *Leuconostoc mesenteroides* (*L. mesenteroides*), a Gram-positive, facultative anaerobic lactic acid bacterium prevalent in traditional fermented foods, such as kimchi, sauerkraut, and miso, has attracted scientific interest because of its heterofermentative metabolism. *L. mesenteroides* metabolizes carbohydrates into lactic acid, ethanol, acetic acid, and carbon dioxide and synthesizes extracellular polysaccharides with prebiotic properties [17]. Preliminary studies have suggested multiple mechanisms underlying the beneficial effects of *L. mesenteroides* on hangover relief, including the enhancement of hepatic–intestinal ADH and ALDH activities to facilitate ethanol and acetaldehyde clearance [18], the reinforcement of intestinal epithelial integrity to reduce alcohol-induced endotoxin-mediated inflammation [19], potent antioxidant activity to mitigate oxidative stress [20], and modulation of the gut–brain axis through interactions with GABAergic and serotonergic pathways to potentially ameliorate alcohol-induced neurobehavioral disturbances [21].

Despite these promising findings, research on the potential of *L. mesenteroides* in mitigating hangover symptoms remains nascent and is primarily limited to in vitro and in vivo studies, with inadequate clinical validation. Therefore, the primary aim of this study was to elucidate the precise molecular mechanisms of *L. mesenteroides* in ethanol metabolism and evaluate its effectiveness in alleviating alcohol-induced symptoms. Moreover, this research seeks to provide scientifically effective strategies for the management and treatment of hangover symptoms.

## 2. Materials and Methods

### 2.1. Study Participants and Inclusion/Exclusion Criteria

Study participants were recruited through an open clinical trial announcement. Initial screening and eligibility assessments were conducted at the Department of Hematology and Oncology, Yonsei University Wonju Severance Christian Hospital (Wonju, Republic of Korea). The screening procedures included a comprehensive physical examination (height and weight), collection of demographic characteristics, detailed medical history, vital signs, assessment of past and concomitant medication use, and urine tests for drug screening and pregnancy (where applicable). All potential participants received a thorough explanation of the study objectives, methodology, potential risks, and participant rights before enrollment. Written informed consent was obtained from each participant prior to their participation. The inclusion and exclusion criteria are listed in Table 1.

### 2.2. Study Design

This study was designed as a randomized, double-blind, placebo-controlled crossover clinical trial to evaluate the effects of *L. mesenteroides* VITA-PB2 on alcohol metabolism and hangover symptoms in healthy adults. This study was conducted between 13 September 2024 and 28 February 2025, after approval from the Institutional Review Board (IRB) of Severance Christian Hospital, Yonsei University Wonju College of Medicine (IRB No. CR324070) (12 September 2024). This trial was prospectively registered at ClinicalTrials.gov (National Clinical Trial Number: NCT07017088). All study procedures strictly adhered to the ethical guidelines stipulated in the Declaration of Helsinki and complied with the Korean Good Clinical Practice (KGCP) standards. After providing written informed consent, participants who fulfilled the study criteria underwent comprehensive clinical evaluation, including physical examinations, measurement of vital signs (blood pressure and pulse rate), anthropometric assessments (height, body weight, and BMI), complete blood count (CBC), and a standardized alcohol consumption questionnaire during visit 1.

The participants were randomly assigned to either the control or intervention group in a 1:1 allocation ratio. Throughout the study period, the participants were instructed to maintain consistent dietary habits and refrain from consuming any foods or dietary supplements that could potentially influence alcohol metabolism. This study employed a double-blind design, wherein both the participants and investigators remained blinded to the group assignments until the completion of all study procedures. During visits 2 and 3, the participants received a single oral dose of either the test product (*L. mesenteroides* VITA-PB2) or placebo, depending on their assigned group. Visit 3 was conducted following a 7-day washout period to eliminate potential carryover effects between interventions.

### 2.3. Interventions

The intervention product used in this study was *L. mesenteroides* VITA-PB2, provided in the form of opaque capsules containing a non-odorous, light-yellow powder. The product was manufactured by Vitabio Inc. (Sejong 30141, Republic of Korea). The placebo, which was visually identical in appearance and form to the intervention product, was 100% maltodextrin. Participants consumed three capsules (either placebo or intervention) in a single dose (total dose of 1.0 × 10^9^ CFU/day) with water. All participants received a standardized meal before trial initiation, and the capsules were administered 30 min after meal ingestion. Subsequently, each participant consumed a predetermined quantity (equivalent to 0.68 g/kg body weight) of the Korean alcoholic beverage soju (20% alcohol by volume), along with shrimp snacks (20 pieces) within 30 min.

### 2.4. Outcome Measurements

#### 2.4.1. Primary Outcomes

The primary outcomes of this study were blood concentrations of ethanol and acetaldehyde and ALDH enzymatic activity as indicators of alcohol metabolism. Venous blood samples were collected at 0, 0.5, 1, 2, and 4 h after alcohol consumption using potassium ethylenediaminetetraacetic acid (EDTA) anticoagulant tubes (BD Biosciences, Franklin Lakes, NJ, USA). At every time point, two blood samples, each 2 mL, were collected from each participant. Blood samples were allowed to coagulate for 15 min, after which the samples were centrifuged. One sample was stored at 4 °C for ethanol concentration and liver function tests, while the other was processed to extract serum and stored at −70 °C for subsequent analysis.

Blood ethanol concentrations were quantified according to standard clinical protocols established by the Department of Laboratory Medicine, Yonsei University Wonju Severance Christian Hospital. The sample handling and transportation complied with the hospital’s internal standard operating procedures to ensure sample integrity. Acetaldehyde concentrations were quantified using the EnzyChrom™ Acetaldehyde Assay Kit (EACT-100, BioAssay Systems, Hayward, CA, USA) following the manufacturer’s protocol. Briefly, 50 μL of serum, consisting of 10 μL of sample and 40 μL of assay buffer, was added into each well of a 96-well microplate. The reaction mixture, comprising the assay buffer, enzyme mix, and chromogenic dye reagent, was subsequently added, and the plate was incubated at room temperature (approximately 22–25 °C) for 30 min. Absorbance was measured at 565 nm using a SpectraMax^®^ ABS Plus microplate reader (Molecular Devices, San Jose, CA, USA). Acetaldehyde levels were determined by interpolating the absorbance values against a standard calibration curve generated from known concentrations. The enzymatic activity of ALDH was assessed using a colorimetric assay kit (Abcam, Cambridge, UK; ab155893). In brief, 50 μL of undiluted serum was added to each well of a 96-well microplate, followed by the addition of the acetaldehyde substrate-containing reaction solution. After 30 min of incubation at room temperature, the absorbance was recorded at 450 nm using a microplate reader (Molecular Devices, San Jose, CA, USA). The increase in optical density was directly proportional to ALDH activity, serving as a quantitative indicator of the catalytic function of the enzyme in acetaldehyde detoxification during ethanol metabolism.

#### 2.4.2. Secondary Outcomes

A survey of hangover severity and symptoms was conducted using the Acute Hangover Scale (AHS) after 4 h of alcohol consumption. The AHS consists of nine items assessing nine hangover symptoms, including discomfort due to hangovers, thirst, fatigue, headache, dizziness, loss of appetite, gastrointestinal disturbances, nausea, and heart palpitations. Each symptom was rated on a 7-point Likert scale ranging from 0 to 7, with standardized anchors: ‘none’ (score = 0), ‘mild’ (score = 1), ‘moderate’ (score = 4), and ‘incapacitating’ (score = 7). The AHS score was calculated as the mean of the nine individual items, providing an overall assessment of hangover severity.

### 2.5. Liver Enzyme Measurements

Serum concentrations of aspartate aminotransferase (AST), alanine aminotransferase (ALT), and gamma-glutamyl transferase (GGT), key biomarkers for evaluating hepatic function and identifying hepatocellular injury, were analyzed at the Department of Laboratory Medicine, Yonsei University Wonju Severance Christian Hospital. Blood samples were collected in potassium EDTA anticoagulant tubes, and serum was separated by centrifugation at 3000 rpm for 10 min. The samples were then transported to the hospital laboratory under refrigerated conditions (4 °C) for immediate analysis. All analytical procedures, including instrument calibration and quality control measures, were rigorously performed according to established internal standard operating procedures.

### 2.6. Oxidative Stress and Antioxidant Defense Assessments

Reactive oxygen species (ROS) and nitric oxide (NO) levels in the serum were measured to evaluate alcohol-induced oxidative stress. ROS levels were quantified using a 2′,7′-dichlorodihydrofluorescein diacetate (DCF-DA) assay (EMD Millipore Corp., Burlington, MA, USA, Cat. #287810-100MG). Briefly, 50 μL of serum was mixed with 100 μL of 20 μM DCF-DA solution in black 96-well plates, followed by incubation at 37 °C for 30 min. The fluorescence intensity was measured using a DTX series multimode plate reader (Beckman Coulter, Fullerton, CA, USA) at excitation/emission wavelengths of 488/525 nm. The NO levels were determined using the Griess Reagent System (Promega, Madison, WI, USA, Cat. #G2930). Equal volumes (50 μL each) of serum and Griess reagent were incubated at room temperature for 10 min in the dark. Subsequently, absorbance was measured at 540 nm using a SpectraMax^®^ ABS Plus microplate reader (Molecular Devices).

To assess the antioxidant capacity, the serum antioxidant glutathione peroxidase (GPx) and catalase enzyme activities and 2,2-diphenyl-1-picrylhydrazyl (DPPH) radical scavenging capacity were evaluated. GPx activity was quantified using a glutathione peroxidase assay kit (Abcam, Cat. #ab102530). In brief, 50 μL of each serum sample was incubated with assay buffer, tert-butyl hydroperoxide, and nicotinamide adenine dinucleotide phosphate (NADPH). Absorbance was measured at 340 nm to monitor NADPH oxidation, and GPx activity was calculated using a standard curve. Catalase activity was measured using a catalase activity kit from Abcam (Abcam, Cat. #ab83464). Diluted serum samples (20 μL) were incubated with the hydrogen peroxide substrate and assay buffer at room temperature for 30 min. After addition of the stop solution and chromogenic reagent, absorbance of the sample was measured at 570 nm, and catalase activity was determined according to the manufacturer’s instructions. The DPPH radical scavenging activity was assessed using a DPPH Antioxidant Assay Kit (Colorimetric; Abcam, Cat. #ab289847). The serum samples (100 μL) were mixed with an equal volume of freshly prepared DPPH working solution in 96-well plates. After samples were incubated at room temperature for 30 min in the dark, absorbance was measured at 517 nm, and antioxidant capacity was expressed as Trolox equivalents in μM, calculated from a standard curve.

### 2.7. Statistical Analysis

Statistical analyses were conducted using the GraphPad Prism software (version 10.1.2; GraphPad Software, La Jolla, CA, USA). Continuous variables are presented as mean ± standard deviation (SD), and categorical variables are presented as frequencies (*n*) and percentages (%). Group comparisons were performed using two-way repeated-measures ANOVA with post hoc multiple comparisons. Statistical significance was set at *p* < 0.05.

## 3. Results

### 3.1. Participant Characteristics

**Figure 1:**
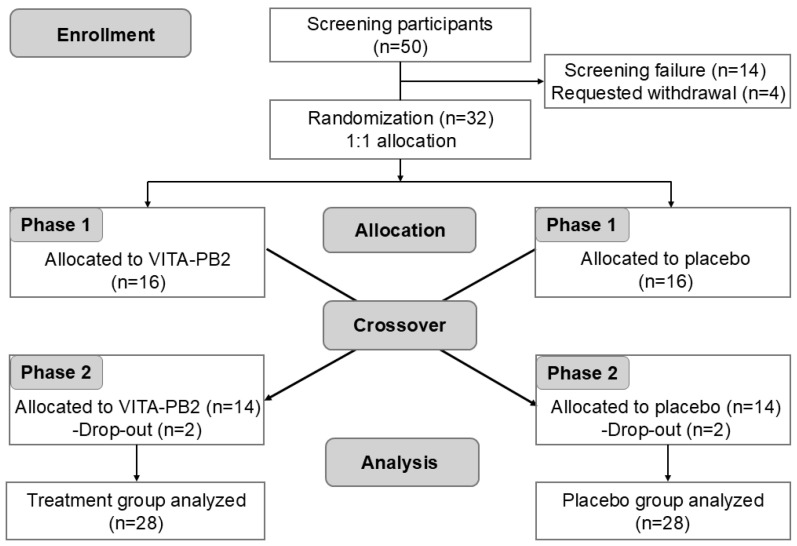
CONSORT flow diagram of participant enrollment, allocation, and analysis.

A total of 50 participants underwent an initial screening process consisting of structured questionnaires, comprehensive physical examinations, and standardized laboratory tests to determine eligibility. Among these participants, 18 were excluded per the inclusion and exclusion criteria. Consequently, 32 participants were enrolled and randomized into this study. According to the crossover design, participants received the opposite treatment after a 7-day washout period. During the trial period, four participants (two from the VITA-PB2 intervention group and two from the placebo group) withdrew their informed consent and discontinued participation. Ultimately, 28 participants completed the trial protocol and were included in the final statistical analysis (Figure 1).

The demographic and baseline characteristics of the participants are summarized in Table 2. The intervention group consisted of six male (42.86%) and eight female (57.14%) participants, whereas the placebo group comprised eight male (57.14%) and six female participants (42.86%). The mean age of the intervention group was 43.07 ± 8.03 years, compared to 45.14 ± 5.68 years in the placebo group, with no significant intergroup differences. Additionally, no significant differences were found between the groups in terms of hematological parameters, such as white blood cell count (WBC), red blood cell count (RBC), hemoglobin (Hb), and platelet count (PLT), body composition metrics (BMI, height, weight, body fat percentage, and basal metabolic rate), or vital signs (systolic and diastolic blood pressure, pulse rate, and body temperature). These results indicate that the participants in both groups were comparable at baseline.

### 3.2. Effect of VITA-PB2 on Blood Alcohol and Acetaldehyde Levels

Table 3 shows the blood ethanol and acetaldehyde concentrations measured at five distinct intervals (0, 0.5, 1, 2, and 4 h) after alcohol ingestion. Although the differences in blood ethanol concentrations between the placebo and VITA-PB2 groups were not statistically significant at any specific time point, the ethanol concentrations in the VITA-PB2 group consistently remained lower than those in the placebo group from 0.5 h onward. The sustained decline in ethanol concentration implies a potential mitigating effect of VITA-PB2 supplementation on blood ethanol accumulation following alcohol consumption.

A significant difference was observed at the 1 h mark in blood acetaldehyde concentrations, wherein the VITA-PB2 group demonstrated a notably lower mean acetaldehyde concentration (3.42 ± 0.38 mg/dL) compared to the placebo group (5.08 ± 1.56 mg/dL; *p* = 0.04), representing a 32.68% reduction. These findings suggest that VITA-PB2 supplementation may enhance acetaldehyde metabolism or attenuate its formation following ethanol exposure.

### 3.3. Effect of VITA-PB2 on Blood ALDH Activity

At 0.5 h post-alcohol consumption, the mean ALDH activity in the VITA-PB2 group (6.08 ± 2.48 mU/mL) was significantly higher than that in the placebo group (4.27 ± 2.40 mU/mL; *p* = 0.03), corresponding to a 42.15% increase (Table 4). Although no significant differences were observed at other time points, the ALDH concentrations in the VITA-PB2 group remained consistently higher than those in the placebo group. Notably, 1 h post-alcohol consumption, the ALDH level in the VITA-PB2 group was 6.16 ± 0.88 mU/mL, representing a 16.23% increase compared to 5.30 ± 1.35 mU/mL in the placebo group.

### 3.4. Effect of VITA-PB2 on AHS Scores After Alcohol Consumption

AHS scores were compared between the placebo and VITA-PB2 groups to assess potential differences in the overall severity and symptoms of hangovers (Table 5). The mean total AHS score in the VITA-PB2 group was 0.44 ± 0.37, representing a 59.63% reduction compared to 1.09 ± 1.18 in the placebo group. Although this difference was not statistically significant, this trend suggests that VITA-PB2 supplementation may alleviate the overall severity of hangovers. Further evaluation of individual AHS indicators revealed consistently lower scores in the VITA-PB2 group for ‘hangovers, fatigue’, ‘headache’, ‘dizziness’, ‘gastrointestinal disturbances’, ‘nausea’, and ‘heart palpitations’ compared to those in the placebo group. In contrast, the ‘loss of appetite’ score was 24.14% higher in the VITA-PB2 group. Importantly, a significant difference was observed for the symptom of ‘thirst’ (*p* = 0.048), with the placebo group reporting an 81.55% higher score than the VITA-PB2 group.

### 3.5. Effect of VITA-PB2 on Liver Enzyme Activity

To assess the effects of VITA-PB2 supplementation on liver enzyme activity following alcohol consumption, the serum levels of AST, ALT, and GGT were measured at 0, 0.5, and 4 h after ingestion (Table 6). There were no statistically significant differences in any liver enzyme parameters between the placebo and VITA-PB2 groups at any time point.

### 3.6. Effect of VITA-PB2 on ROS and NO Levels

Alcohol metabolism induces ROS production and interferes with NO signaling pathways, thereby triggering systemic oxidative stress. To evaluate the potential protective effects of VITA-PB2 supplementation against alcohol-induced oxidative damage, the dynamic changes in blood ROS and NO levels were assessed. Given the transient nature and high reactivity of NO, its stable metabolite, nitrite (NO_2_^−^), is commonly used as a reliable surrogate marker to reflect endogenous NO levels in vivo.

As shown in Table 7, both the placebo and VITA-PB2 groups exhibited increased ROS levels following alcohol consumption. However, at the 1 h post-ingestion time point, the ROS levels in the VITA-PB2 group (704.17 ± 107.90) were significantly lower than those in the placebo group (797.44 ± 91.46, *p* = 0.02), suggesting that VITA-PB2 may exert acute antioxidant effects. At 2 and 4 h post-consumption, ROS levels in both groups returned to near-baseline measurements. Regarding NO metabolism, no significant differences in blood NO levels were observed between the two groups at any time point. Nonetheless, a transient increase in NO was noted in the placebo group (0.34 ± 0.17 μM) at 1 h post-alcohol intake, with levels 35.29% higher than those in the VITA-PB2 group (0.22 ± 0.17 μM). Throughout all time points, the VITA-PB2 group maintained relatively lower NO levels. Collectively, these results suggest that VITA-PB2 has antioxidant potential, especially for rapidly alleviating oxidative stress following acute alcohol exposure.

### 3.7. Effect of VITA-PB2 on Antioxidant Enzyme Activities and Free Radical Scavenging Capacity

To further investigate the potential antioxidant effects of VITA-PB2 supplementation, we measured changes in blood catalase and GPx activities, as well as DPPH radical scavenging capacity (Table 8), within 0–4 h after alcohol intake, to value enzymatic and non-enzymatic antioxidant defense systems. The results showed that no significant differences in catalase activity were observed between the placebo and VITA-PB2 groups at any time point. However, at 1 h post-intake, catalase activity in the VITA-PB2 group (28.59 ± 4.13 mU/mL) increased notably and was 24.41% higher than that in the placebo group (22.98 ± 6.10 mU/mL). In contrast, the GPx activity exhibited more pronounced intergroup differences. No notable changes were detected during the 1 h after alcohol intake; however, at 2 h, GPx activity in the VITA-PB2 group peaked at 431.58 ± 149.50 mU/mL, which was significantly higher than that in the placebo group (315.19 ± 39.81 mU/mL, *p* = 0.002). The elevated GPx levels were maintained for 4 h, indicating that VITA-PB2 may alleviate oxidative stress by enhancing GPx-mediated antioxidant defenses. Furthermore, the DPPH radical scavenging activity (% inhibition) in the VITA-PB2 group began to increase at 1 h post-intake and remained consistently higher than that in the placebo group throughout the observation period. Although no significant differences were observed at any time point, the overall trend suggests that VITA-PB2 supplementation may help improve the non-enzymatic radical scavenging capacity in the blood.

## 4. Discussion

This randomized, double-blind, placebo-controlled crossover trial systematically evaluated the modulatory effects of the probiotic *L. mesenteroides* VITA-PB2 on human ethanol metabolism and hangover-related outcomes. In a previous study, VITA-PB2 exhibited superior characteristics among lactic acid bacterial candidates, including substantial alcohol tolerance, physiological stability, and enhanced metabolic capacity [22]. Ethanol tolerance has also been identified as an essential trait for *Lactobacillus plantarum* strains, particularly those inhabiting ethanol-rich environments such as wine and beer. Recent research indicates that the regulator AcrR modulates fatty acid biosynthesis pathways, facilitating the adaptation of *L. plantarum* strains to toxic ethanol levels [23]. Additionally, studies have shown that *Lactococcus lactis* significantly improved alcohol metabolism in mice by reducing blood alcohol concentrations, enhancing ADH activities in stomach and liver tissues, and alleviating alcohol-induced liver damage, further illustrating probiotic-mediated protection against ethanol toxicity [24]. Furthermore, *Lactobacillus rhamnosus* GG supplementation has been shown to ameliorate alcohol-induced liver injury through mechanisms involving reduction in gut-derived endotoxemia and the restoration of intestinal epithelial barrier function [25]. Additionally, studies involving rodent models subjected to acute alcohol exposure indicated that supplementation with VITA-PB2 significantly elevated hepatic enzyme activity, reduced circulating ethanol and acetaldehyde concentrations, and mitigated oxidative stress and associated tissue injury [14]. These findings suggested that VITA-PB2 possesses notable bioactivity in vivo, highlighting its potential therapeutic implications in alcohol metabolism and related physiological protection.

The dietary intake of specific nutrients and components has been shown to influence ethanol metabolism, subsequently affecting the severity and occurrence of hangover symptoms [26]. A higher intake of dietary nutrients, such as niacin and zinc, has been associated with reduced severity of hangovers [27], and the consumption of beverages, including soda water, green tea, and honey chrysanthemum tea, has been shown to enhance ethanol clearance and mitigate alcohol-induced hepatic injury when consumed concomitantly with excessive alcohol [28]. These findings highlight the critical role of dietary variation in hangover outcomes. Therefore, this study standardized the participants’ dietary conditions before trial initiation. All participants consumed uniform meals to minimize the potential confounding influence of diet on study outcomes.

Ethanol metabolism in humans predominantly involves sequential enzymatic oxidation reactions catalyzed by ADH and ALDH, whereby ethanol is initially converted into acetaldehyde within the cytosol, followed by its subsequent transformation into acetate within the mitochondria [29]. Impaired ethanol metabolism is a significant pathogenic factor underlying alcohol hangover [9]. Moreover, faster ethanol elimination has been associated with a reduced severity and incidence of hangover symptoms [30,31]. This study employed serial pharmacokinetic assessments of blood ethanol and acetaldehyde concentrations, along with ALDH activity measurements to comprehensively evaluate the modulatory effects of VITA-PB2 supplementation on these metabolic pathways. Although intergroup differences in blood ethanol concentrations at discrete time intervals did not achieve statistical significance, the ethanol concentrations in the VITA-PB2 group remained lower than those in the placebo group from 0.5 h onward. These findings suggest that enhanced ethanol clearance, or a possible influence on ethanol bioavailability, is associated with VITA-PB2 supplementation.

Acetaldehyde, as the primary intermediate metabolite formed during ethanol oxidation, exhibits markedly greater toxicological properties than ethanol itself and is widely recognized as a pivotal mediator of alcohol-induced adverse effects [32,33,34]. In this study, at 1 h post-ethanol consumption, an approximate 33% reduction in blood acetaldehyde concentrations was observed in participants receiving VITA-PB2 compared with those receiving placebo. These findings provide evidence that VITA-PB2 supplementation significantly facilitates ethanol detoxification and reduces systemic acetaldehyde exposure; thus, VITA-PB2 holds substantial therapeutic potential for alleviating the adverse physiological outcomes associated with acute alcohol consumption.

As the rate-limiting enzyme governing acetaldehyde metabolism, ALDH modulates both the duration and magnitude of systemic acetaldehyde exposure and, therefore, the associated toxicological consequences [7,35,36]. In the present study, serum ALDH activity was significantly elevated at 0.5 h post-alcohol ingestion in participants receiving VITA-PB2 supplementation, reflecting an increase of 42.15% compared to the placebo group. This enhancement persisted across subsequent measurement intervals, indicating the sustained upregulation of ALDH activity. Importantly, temporal elevations in serum ALDH activity inversely correspond to reductions in circulating acetaldehyde concentrations, substantiating the mechanistic role of ALDH in accelerating acetaldehyde detoxification [37]. Mitochondrial ALDH2, the primary isoform responsible for acetaldehyde detoxification, frequently exhibits genetic polymorphisms, notably the ALDH2*2 allele (Lys487) that is prevalent in East Asian populations. These polymorphisms significantly reduce enzymatic activity, impair acetaldehyde clearance, and consequently increase the susceptibility to alcohol-induced adverse effects, including pronounced hangover symptoms [38,39]. Therefore, enhancing ALDH activity using dietary compounds or probiotics has emerged as a promising strategy for mitigating alcohol-related toxicity.

Alcohol clearance rates in humans exhibit considerable variability and are influenced by factors such as sex, hepatic enzyme function, BMI, and dietary status. Nonetheless, the alignment among reduced ethanol concentrations, elevated ALDH activity, and significantly decreased acetaldehyde levels in participants supplemented with VITA-PB2 collectively highlights the modulatory effect of VITA-PB2 on key pathways involved in alcohol metabolism. Subjective evaluation using the AHS revealed a mean total score of 0.44 in the VITA-PB2 group, reflecting a 59.63% reduction relative to the placebo group. Notably, individual symptom scores, including fatigue, headache, dizziness, gastrointestinal disturbances, nausea, heart palpitations, and general hangover severity, were consistently lower among participants receiving VITA-PB2. Of particular note, thirst scores were reduced by nearly 82% in the VITA-PB2 group compared to the placebo group. Thirst, commonly intensified by acetaldehyde neurotoxicity and fluid loss associated with ethanol metabolism [40], corresponded closely with the observed reduction in acetaldehyde concentrations, suggesting that VITA-PB2 may mitigate discomfort through the accelerated detoxification of harmful metabolites. Emerging evidence indicates that certain lactic acid bacterial strains can ameliorate alcohol-related hepatic injury by strengthening intestinal epithelial barrier integrity, modulating gut microbiota composition, and suppressing pro-inflammatory cytokine expression [41,42,43]. In the present study, serum levels of hepatic enzymes (AST, ALT, and GGT) measured 0.5 and 4 h after alcohol ingestion exhibited no significant differences between the placebo and VITA-PB2 groups. This finding suggests that acute alcohol exposure in otherwise healthy individuals may not induce sufficiently pronounced elevations in hepatic enzymes, thus potentially obscuring the probiotic-mediated hepatoprotective effects in this study. Nevertheless, the absence of acute hepatic enzyme changes does not exclude the possibility that VITA-PB2 supplementation confers beneficial effects on chronic or high-dose alcohol consumption.

Alcohol metabolism generates the toxic intermediate acetaldehyde and simultaneously stimulates ROS production via the activation of cytochrome P450 enzymes and mitochondrial electron transport chains. It also simultaneously disrupts NO signaling pathways, collectively resulting in systemic oxidative stress [44,45,46]. In the present study, changes in circulating ROS and NO levels and antioxidant enzyme activities were monitored to comprehensively evaluate the antioxidative potential of VITA-PB2 following acute alcohol ingestion. Both groups exhibited elevated ROS levels at 0.5 h post-ingestion. However, at 1 h, the VITA-PB2 group demonstrated significantly lower ROS concentrations, representing an 11.70% reduction compared to the placebo group. This observation suggests a rapid attenuation of the initial alcohol-induced oxidative burden mediated by VITA-PB2. Although differences in NO levels were not statistically significant, a 35.29% lower concentration was observed in the VITA-PB2 group at the 1 h mark, potentially reflecting regulatory effects on NO metabolism.

The antioxidative properties of probiotics have attracted considerable scientific attention, encompassing the direct scavenging of oxidants, such as hydrogen peroxide and superoxide anions, and the modulation of host antioxidant enzymes. Previous investigations have demonstrated that various lactic acid bacteria, including VITA-PB2, significantly enhance the host activities of GPx, superoxide dismutase, and catalase, thereby fortifying systemic antioxidative defense [47,48,49]. Moreover, fermentation-derived metabolites, including short-chain fatty acids, glutathione, exopolysaccharides, and bioactive peptides, contribute additional antioxidative effects through intestinal epithelial repair, modulation of the Nrf2 signaling pathway, and the inhibition of NADPH oxidase activity [50,51,52]. The present study demonstrates that catalase activity at 1 h post-ethanol ingestion increased by 24.41% in the VITA-PB2 group compared to placebo, whereas GPx activity was significantly higher at 2 h, with this elevation sustained at the 4 h time point. These findings indicated a prolonged enzymatic antioxidative response following the initial suppression of ROS. Additionally, the DPPH radical scavenging activity (% inhibition), which is indicative of the non-enzymatic total antioxidant capacity, consistently increased in the VITA-PB2 group throughout the observation period, suggesting a biologically relevant antioxidative contribution.

Despite these promising results, this study had some limitations. The small sample size and short intervention period may limit the generalizability of our findings. Future studies should include larger and more demographically diverse populations to validate and extend our results. The long-term effects and safety of VITA-PB2 remain unclear and warrant extended follow-up trials to assess its sustained effects on alcohol metabolism and related physiological parameters. In addition, genetic polymorphisms in alcohol-metabolizing enzymes (e.g., ALDH2 and ADH1B) significantly influence individual variability in alcohol response. Stratifying participants by genotype may provide a more precise and personalized evaluation of VITA-PB2 efficacy. Finally, investigating the possible synergistic effects of VITA-PB2 with other probiotic strains or prebiotics may further enhance its therapeutic potential as a part of a broader microbiome-targeted strategy.

## 5. Conclusions

In summary, *L. mesenteroides* VITA-PB2 enhances ALDH activity, promotes acetaldehyde clearance, and reduces ethanol-induced toxicity. Uniquely compared with other tested strains, it sustains antioxidant enzyme activation, lowers ROS levels, and effectively relieves hangover symptoms like thirst, emphasizing its potential as a functional probiotic against alcohol-related stress.

## Figures and Tables

**Table 1:** Inclusion and exclusion criteria for study participants.

Inclusion Criteria	Exclusion Criteria
Healthy adults aged 30–60 years. Body mass index (BMI) between 18.5 kg/m^2^ and 30 kg/m^2^. History of experiencing hangover symptoms. Normal blood test results and vital signs. No organic gastrointestinal diseases confirmed using gastroscopy within the past three months. Ability to consume Korean alcoholic beverage soju (20% alcohol by volume) at a dose equivalent to 0.68 g/kg body weight within 30 min. Written informed consent.	History of excessive alcohol consumption within the preceding week. Participation in other clinical trials within the past month. Use of dietary supplements or medications that may influence alcohol metabolism. Diagnosed alcohol metabolism disorders, diabetes, hypertension, cholelithiasis, pancreatitis, gout, active tuberculosis, gastrointestinal bleeding, or surgery. Renal or hepatic diseases (e.g., hepatitis B or C, alcoholic liver disease), cardiovascular, pulmonary, gastrointestinal, hepatic, or neurological disorders. Pregnancy or plans to conceive during the study period. Use of liver function-enhancing medications or supplements within one month before study initiation. Excessive alcohol intake within one week after trial participation. Any other medical or psychological condition deemed unsuitable for study participation by the investigators.

**Table 2:** General characteristics of the participants at baseline.

Variables		Total Participants (*n* = 28)
	Age (years)	44.11 ± 6.91
Sex	Male	14
	Female	14
Hematological parameters	WBC (10^3^/µL)	5.83 ± 1.25
	RBC (10^6^/µL)	4.75 ± 0.31
	Hb (g/dL)	14.50 ± 1.21
	PLT (10^3^/µL)	269.07 ± 68.26
Body composition metrics	Height (cm)	167.46 ± 8.76
	Weight (kg)	69.51 ± 13.78
	BMI (kg/m^2^)	24.61 ± 3.32
	Body Fat (%)	27.95 ± 8.09
	Basal Metabolic Rate (kcal)	1452.89 ± 244.21
Vital signs	Systolic Blood Pressure (mm/Hg)	109.29 ± 10.16
	Diastolic Blood Pressure (mm/Hg)	70.71 ± 7.16
	Pulse (beats per min)	74.43 ± 4.12
	Temperature (°C)	36.44 ± 0.15

Values are presented as mean ± SD or number. WBC, white blood cell count; RBC, red blood cell count; Hb, hemoglobin; PLT, platelet count; and BMI, body mass index.

**Table 3:** Effects of VITA-PB2 on blood alcohol and acetaldehyde concentrations following alcohol consumption.

Parameter	Time Post-Alcohol Consumption (h)	Placebo (*n* = 28)	VITA-PB2 (*n* = 28)	*p*-Value ^1^
Blood alcohol level (mg/dL)	0	0.12 ± 0.33	0.72 ± 1.79	1.00
	0.5	54.20 ± 31.82	46.71 ± 18.81	0.82
	1	56.22 ± 23.49	49.18 ± 18.25	0.85
	2	53.72 ± 20.92	49.70 ± 17.11	0.99
	4	35.11 ± 16.62	30.95 ± 12.74	0.98
Blood acetaldehyde level (mg/dL)	0	3.23 ± 1.19	3.24 ± 1.64	1.00
	0.5	3.25 ± 1.19	3.47 ± 1.44	1.00
	1	5.08 ± 1.56	3.42 ± 0.38	0.04 *
	2	4.63 ± 0.83	4.11 ± 0.53	0.97
	4	4.67 ± 1.50	3.42 ± 0.78	0.31

Values are presented as mean ± SD. ^1^ Statistical analysis is performed using a two-way repeated-measures ANOVA with post hoc multiple comparisons (compared between the placebo and VITA-PB2 groups). Statistically significant differences between groups are indicated by * *p* < 0.05.

**Table 4:** Effects of VITA-PB2 on blood ALDH activity following alcohol consumption.

Parameter	Time Post-Alcohol Consumption (h)	Placebo (*n* = 28)	VITA-PB2 (*n* = 28)	*p*-Value ^1^
ALDH (mU/mL)	0	3.14 ± 0.85	3.73 ± 0.57	0.94
	0.5	4.27 ± 2.40	6.08 ± 2.48	0.03 *
	1	5.30 ± 1.35	6.16 ± 0.88	0.87
	2	4.58 ± 0.92	4.97 ± 1.81	0.99
	4	4.12 ± 1.42	4.60 ± 1.13	0.95

Values are presented as mean ± SD. ALDH, acetaldehyde dehydrogenase. ^1^ Statistical analysis is performed using two-way repeated-measures ANOVA with post hoc multiple comparisons (compared between the placebo and VITA-PB2 groups). Statistically significant differences between groups are indicated by * *p* < 0.05.

**Table 5:** Mean AHS score and individual hangover symptom scores.

Indicators	Placebo (*n* = 28)	VITA-PB2 (*n* = 28)	*p*-Value ^1^
Hangover	1.43 ± 2.17	0.86 ± 1.10	0.99
Thirst	2.71 ± 2.30	0.50 ± 0.65	0.048 *
Fatigue	3.07 ± 2.59	1.50 ± 1.40	0.64
Headache	0.50 ± 1.09	0.14 ± 0.36	0.97
Dizziness	0.36 ± 0.84	0.21 ± 0.58	1.00
Loss of appetite	0.29 ± 0.73	0.36 ± 0.84	1.00
Gastrointestinal disturbances	0.43 ± 1.34	0.14 ± 0.36	1.00
Nausea	0.43 ± 1.09	0.14 ± 0.36	1.00
Heart palpitations	0.57 ± 1.09	0.07 ± 0.27	0.77
Mean AHS score	1.09 ± 1.18	0.44 ± 0.37	0.31

Values are presented as mean ± SD. AHS, Acute Hangover Scale. ^1^ Statistical analysis is performed using the Wilcoxon matched-pair signed-rank test (compared between the placebo and VITA-PB2 groups). Statistically significant differences between groups are indicated by * *p* < 0.05.

**Table 6:** Effects of VITA-PB2 on liver enzyme activity following alcohol consumption.

Liver Enzyme	Time Post-Alcohol Consumption (h)	Placebo (*n* = 28)	VITA-PB2 (*n* = 28)	*p*-Value ^1^
AST (U/L)	0	23.07 ± 5.73	20.86 ± 6.80	0.80
	0.5	27.86 ± 6.36	27.67 ± 11.00	1.00
	4	27.36 ± 5.89	25.43 ± 6.12	0.86
ALT (U/L)	0	17.40 ± 4.84	17.21 ± 8.75	1.00
	0.5	16.80 ± 5.03	18.71 ± 8.02	0.87
	4	16.40 ± 4.45	16.79 ± 6.44	1.00
GGT (U/L)	0	18.90 ± 6.37	17.67 ± 8.64	0.97
	0.5	18.50 ± 5.08	19.50 ± 9.81	0.98
	4	18.40 ± 5.30	16.75 ± 5.75	0.93

Values are presented as mean ± SD. AST, aspartate aminotransferase; ALT, alanine aminotransferase; and GGT, gamma-glutamyl transferase. ^1^ Statistical analysis is performed using two-way repeated-measures ANOVA with post hoc multiple comparisons (compared between the placebo and VITA-PB2 groups).

**Table 7:** Effects of VITA-PB2 on blood ROS and NO levels following alcohol consumption.

Parameter	Time Post-Alcohol Consumption (h)	Placebo (*n* = 28)	VITA-PB2 (*n* = 28)	*p*-Value ^1^
ROS level (Fluorescence Intensity)	0	718.05 ± 31.66	706.98 ± 97.46	1.00
	0.5	763.71 ± 96.11	782.59 ± 72.54	0.98
	1	797.44 ± 91.46	704.17 ± 107.90	0.02 *
	2	681.48 ± 20.43	659.18 ± 61.25	0.99
	4	758.66 ± 48.10	759.70 ± 30.56	1.00
NO_2_^−^ (μM)	0	0.23 ± 0.13	0.20 ± 0.11	1.00
	0.5	0.28 ± 0.24	0.22 ± 0.14	0.95
	1	0.34 ± 0.17	0.22 ± 0.17	0.38
	2	0.27 ± 0.14	0.21 ± 0.15	0.97
	4	0.25 ± 0.21	0.19 ± 0.13	0.96

Values are presented as mean ± SD. ROS, reactive oxygen species; NO, nitric oxide. ^1^ Statistical analysis is performed using two-way repeated-measures ANOVA with post hoc multiple comparisons (compared between the placebo and VITA-PB2 groups). Statistically significant differences between groups are indicated by * *p* < 0.05.

**Table 8:** Effects of VITA-PB2 on blood catalase, GPx activities, and DPPH (% inhibition) following alcohol consumption.

Parameter	Time Post-Alcohol Consumption (h)	Placebo (*n* = 28)	VITA-PB2 (*n* = 28)	*p*-Value ^1^
Catalase (mU/mL)	0	32.07 ± 4.54	29.60 ± 3.91	0.94
	0.5	26.70 ± 6.90	25.79 ± 7.56	1.00
	1	22.98 ± 6.10	28.59 ± 4.13	0.36
	2	24.14 ± 7.02	24.60 ± 8.00	1.00
	4	26.63 ± 5.32	25.93 ± 4.38	1.00
GPx (mU/mL)	0	246.36 ± 33.31	281.02 ± 68.76	0.66
	0.5	274.60 ± 39.82	269.04 ± 31.70	1.00
	1	259.12 ± 40.07	263.60 ± 46.24	1.00
	2	315.19 ± 39.81	431.58 ± 149.50	0.002 **
	4	348.15 ± 49.45	391.93 ± 132.11	0.70
DPPH (% inhibition)	0	55.75 ± 9.13	53.84 ± 16.54	1.00
	0.5	53.69 ± 10.65	53.55 ± 6.75	1.00
	1	52.45 ± 7.32	60.26 ± 15.83	0.45
	2	50.92 ± 10.87	59.26 ± 8.23	0.44
	4	56.51 ± 9.59	63.30 ± 7.64	0.74

Values are presented as mean ± SD. GPx, glutathione peroxidase; DPPH, 2,2-diphenyl-1-picrylhydrazyl. ^1^ Statistical analysis is performed using two-way repeated-measures ANOVA with post hoc multiple comparisons (compared between the placebo and VITA-PB2 groups). Statistically significant differences between groups are indicated by ** *p* < 0.01.

## Data Availability

The original contributions presented in this study are included in the article. Further inquiries can be directed to the corresponding authors.

## Abbreviations

The following abbreviations are used in this manuscript:

ADH	Alcohol dehydrogenase
ALDH	Acetaldehyde dehydrogenase
CFUs	Colony-forming units
BMI	Body mass index
CBC	Complete blood count
AHS	Acute Hangover Scale
AST	Aspartate aminotransferase
ALT	Alanine aminotransferase
GGT	Gamma-glutamyl transferase
ROS	Reactive oxygen species
NO	Nitric oxide
DCF-DA	2′,7′-dichlorodihydrofluorescein diacetate
GPx	Glutathione peroxidase
DPPH	2,2-diphenyl-1-picrylhydrazyl
NADPH	Nicotinamide adenine dinucleotide phosphate
SD	Standard deviation
ANOVA	Analysis of variance
WBC	White blood cell count
RBC	Red blood cell count
Hb	Hemoglobin
PLT	Platelet count
H	Hour
NO_2_^−^	Nitrite
